# Nosocomial meningitis associated with external ventricular drainage at neurosurgical intensive care unit

**DOI:** 10.1186/2047-2994-4-S1-P247

**Published:** 2015-06-16

**Authors:** N Kurdyumova, GV Danilov, O Ershova, M Shifrin, I Savin, I Alexandrova, E Sokolova

**Affiliations:** 1 burdenko Neurosurgery Institute, Moscow, Russian Federation

## Introduction

Nosocomial meningitis (NM) is a life-threatening complication following neurosurgery. Risk factors for NM are a subject for rigorous investigation.

## Objectives

Our aim was to study the impact of external ventricular drainage (EVD) on NM incidence at neurosurgical intensive care unit (NICU).

## Methods

A prospective surveillance of nosocomial infections at NICU was conducted in 2010 - 2014. Data on 1749 patients stayed at NICU for longer than 48 hours were daily collected into electronic medical records system. Definitions of nosocomial infections developed by Centers for Disease Control and Prevention (CDC, USA) were used as criteria for meningitis diagnosis. External ventricular drainage (EVD) was placed before the manifestation of NM in 404 patients, which were selected for the analysis.

## Results

EVD-associated meningitis (EVDAM) occurred in 89 patients representing 22,0 ± 2,1 (CI 18.0 - 26.2) per 100 patients with EVD. The incidence of EVDAM per 1000 days of EVD functioning was 17.3 ‰. The incidence of meningitis in all patients treated at NICU longer than 48 hours was 12.6% ± 1.0% (CI 10.74% - 14.66%). The relative risk (RR) of meningitis associated with EVD was 4.4. Patients with EVDAM did not differ significantly by gender, age and comorbidity from the rest of patients with EVD (p > 0,05). CSF leakage was more common in patients with EVDAM (14.6% vs. 5.4%, p < 0,05). NM developed within 6 days after EVD placement in 50% of cases, 75% of NM cases occurred within 11 days. The etiology of NM was verified in 62.7% of cases. Coagulase-negative staphylococci (26.0%) and Acinetobacter baumannii (21.0%) were the main pathogens.

## Conclusion

EVD was a significant risk factor for NM leading to 4.4-fold increase in NM incidence at neurosurgical intensive care unit.

## Disclosure of interest

None declared.

